# Role of long non-coding RNAs in the regulation of ferroptosis in tumors

**DOI:** 10.3389/fimmu.2025.1568567

**Published:** 2025-03-21

**Authors:** Ying Ju, Yuanhao Lv, Xu Liu, Jing Lu, Yashen Shi, Huimin Guo, Siguang Xu, Jiaqi Tian, Jun Yang, Jiateng Zhong

**Affiliations:** ^1^ Department of Gynecology, The First Affiliated Hospital of Xinxiang Medical University, Xinxiang, China; ^2^ Department of Pathology, The First Affiliated Hospital of Xinxiang Medical University, Xinxiang, China; ^3^ Department of Pathology, School of Basic Medical Sciences, Xinxiang Medical University, Xinxiang, China; ^4^ Department of Anesthesia and Perioperative Medicine, The First Affiliated Hospital of Xinxiang Medical University, Xinxiang, China; ^5^ Xinxiang Engineering Technology Research Center of Digestive Tumor Molecular Diagnosis, the First Affiliated Hospital of Xinxiang Medical University, Xinxiang, China

**Keywords:** long non-coding RNA, tumor, ferroptosis, drug resistance, tumor therapy

## Abstract

Normal cells begin to grow indefinitely and immortalize to form tumor cells after an external stimulus resulting in a genetic mutation. Effective killing of tumor cells is the basis of various cancer therapies. Ferroptosis is a class of cell death types dependent on iron and cellular lipid peroxidation. Tumors themselves are iron-dependent, and conventional radiotherapy also sensitizes cancer cells to ferroptosis. Increasing the sensitivity of tumor cells to ferroptosis may be a potential therapeutic strategy to overcome the resistance mechanisms of conventional cancer therapy. Long noncoding RNAs (LncRNAs) are a class of transcripts more than 200 nucleotides in length that regulate gene expression at multiple levels and are involved in biological processes such as cell differentiation, cell cycle arrest, and maintenance of tumor stemness. Recent studies have found that lncRNAs regulate ferroptosis of tumor cells through multiple mechanisms and may influence or ameliorate tumor resistance to chemotherapeutic agents. With the continuous maturation of nanomaterials technology, it may provide new means for cancer treatment by regulating the levels of ferroptosis-related lncRNAs inside tumors as well as increasing the levels of Fe^2+^ and ROS inside tumors. In this paper, we systematically introduce the regulatory mechanism of lncRNAs in ferroptosis, the role of ferroptosis in tumor immunotherapy and the application of lncRNAs combined with ferroptosis in nanomaterials, which provides new perspectives for tumor therapy.

## 1 Introduction

Long noncoding RNAs (lncRNAs) are a class of non-protein-coding transcripts greater than 200 nucleotides in length that play crucial roles in regulating gene expression and various biological processes, including cell differentiation, proliferation, and apoptosis (1). In recent years, lncRNAs have emerged as significant regulators in cancer development and progression, influencing tumor cell proliferation, invasion, and drug resistance (2). They can act as oncogenes or tumor suppressors, depending on the context, and are involved in multiple signaling pathways and cellular mechanisms (3).

Ferroptosis, a novel form of programmed cell death characterized by iron-dependent lipid peroxidation, has garnered significant attention for its potential in cancer therapy (4). Unlike apoptosis, necrosis, or autophagy, ferroptosis is uniquely regulated by cellular iron and lipid metabolism (5). Tumor cells, which often exhibit increased iron dependency and altered lipid metabolism, are particularly susceptible to ferroptosis induction (6). Ferroptosis is triggered by the depletion of glutathione (GSH) and the inactivation of glutathione peroxidase 4 (GPX4), leading to the accumulation of lipid peroxides and ultimately cell death (7). The potential of ferroptosis in tumor treatment lies in its ability to selectively target cancer cells while sparing normal cells. This selective vulnerability can be exploited to develop new therapeutic strategies that overcome resistance to conventional treatments. Moreover, recent studies have shown that ferroptosis can be modulated by various factors, including lncRNAs, which play crucial roles in regulating gene expression and cellular processes. Understanding the mechanisms by which lncRNAs regulate ferroptosis and their interplay with the immune system could provide new insights into enhancing the efficacy of cancer immunotherapy.

Recent studies have revealed that lncRNAs play a pivotal role in regulating ferroptosis in tumor cells. These lncRNAs can influence ferroptosis through multiple mechanisms, including direct interaction with DNA, RNA, or proteins, and by acting as molecular signals, decoys, or scaffolds (8, 9). For instance, lncRNA RP11-89 has been shown to enhance ferroptosis by silencing miR-129-5p expression, leading to increased iron accumulation and lipid peroxidation (10). Additionally, lncRNA LINC00618 interacts with lymphoid-specific helicase (LSH), inhibiting the expression of SLC7A11 and inducing ferroptosis (11). Conversely, lncRNA OIP5-AS1 attenuates ferroptosis in prostate cancer cells by upregulating SLC7A11 expression through the miR-128-3p axis (12).

LncRNAs also modulate ferroptosis by influencing the expression and activity of key proteins involved in lipid metabolism and antioxidant defense (13). For example, lncRNA LINC01134 enhances GPX4 expression by facilitating the binding of nuclear factor NRF2 to the GPX4 promoter, thereby mitigating ferroptosis (14). Furthermore, lncRNA HEPFAL promotes the ubiquitination of SLC7A11, reducing its stability and inducing ferroptosis in hepatocellular carcinoma cells (15). These findings suggest that lncRNAs play a dual role in the regulation of iron death in tumor cells by targeting core ferroptosis pathways (e.g., SLC7A11-GPX4 axis, lipid peroxidation regulatory network) - both promoting and inhibiting, depending on their target of action and molecular mechanism.

Although the relationship between lncRNAs and ferroptosis has been gradually revealed, the complexity of their regulatory networks remains to be deeply resolved. For example, hypoxic conditions in the tumor microenvironment induce lncRNA PMAN expression, which stabilizes SLC7A11 mRNA by promoting nucleoplasmic translocation of the RNA-binding protein ELAVL1 and ultimately inhibits iron death in peritoneal metastasis of gastric cancer (16). In addition, lncRNAs can also affect the stability of ferroptosis-related proteins by modulating protein post-translational modifications (e.g., ubiquitination, palmitoylation) (3), e.g., DUXAP8 promotes resistance to sorafenib in hepatocellular carcinoma by enhancing the palmitoylation modification of SLC7A11 and reducing its lysosomal degradation (17). These mechanisms suggest that targeting specific lncRNAs may be an effective strategy to reverse ferroptosis resistance.

Understanding the intricate relationship between lncRNAs and ferroptosis is crucial for developing new therapeutic strategies to overcome drug resistance and improve cancer treatment outcomes (18). In this review, we will discuss the role of lncRNAs in regulating ferroptosis and their impact on immune responses, with a focus on the potential applications in cancer immunotherapy. We aim to highlight the synergistic effects of ferroptosis induction and immunotherapy, as well as the potential of lncRNAs as therapeutic targets to overcome resistance and improve treatment outcomes.

## 2 Role and mechanisms by which lncRNAs regulate ferroptosis

**Figure 1:**
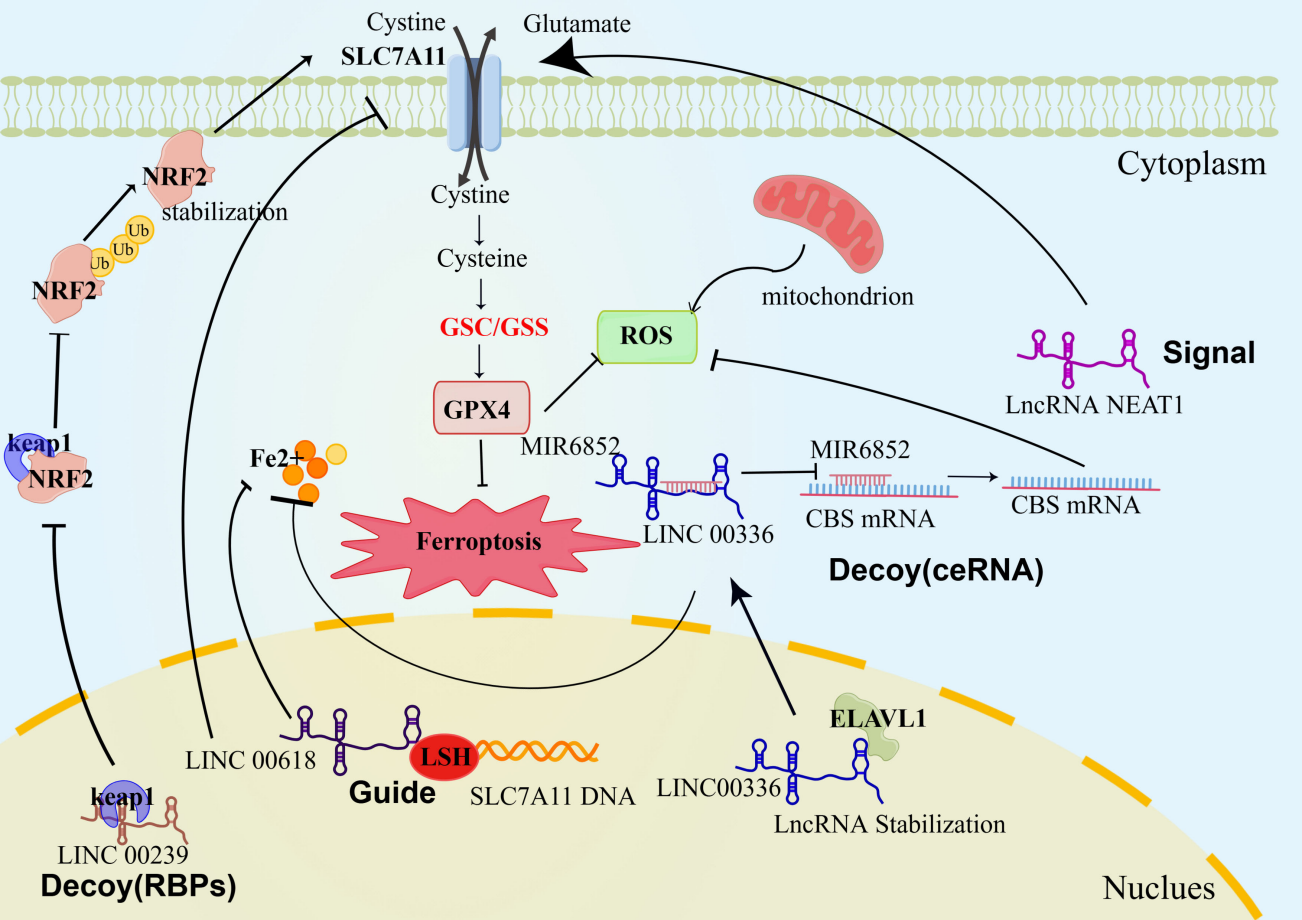
Role of LncRNAs in regulating ferroptosis. 1) As a signal, LncRNA NEAT1 directly promotes SCL7A11 expression and inhibits ferroptosis. 2)As a molecular blocker: ceRNA mech-anism, LINC 00336 binds to MIR6852, leading to a reduction in CBS mRNA degradation and in-hibition of ROS production; RBP mechanism: after binding to keap1, LINC 00239 inhibits the binding of keap1 to NRF2, reduces the ubiquitination of NRF2, stabilises the function of NRF2 and inhibits ferroptosis. 3)Bootstrap mode, the transcribed LncRNAs bind to binding proteins, and the complexes of LncRNAs and proteins are localised to the DNA sequences of the downstream genes LINC00618 binds to LSH and inhibits the transcription of SLC7A11, thus further promoting ferroptosis. LncRNA, long noncoding RNA; RBPs, RNA-binding proteins; LSH, lymphoid-specific helicase; ROS, reactive oxygen species; GPX4, glutathione peroxidase 4; SCL7A11, cystine-glutamate exchange system XC-; CBS, cystathionine beta-synthase; ELAVL1, ELAV-like RNA-binding protein 1; NRF2, nuclear factor-E2-related factor 2; keap1, Kelch-like ECH-related protein 1; Ub, ubiquitination.

LncRNAs regulate ferroptosis through several mechanisms, including direct binding to DNA, proteins, and RNAs, acting as ceRNAs, molecular blockers, and scaffolds (19). **Figure 1** and **Table 1** summarize these four mechanisms.

**Table 1:** Role and mechanisms by which lncRNAs regulate ferroptosis.

Mechanism	Description	Example
Signaling Function	LncRNAs directly regulate the transcription of downstream genes (20).	Lnc NEAT1 binds to and inhibits SLC7A11 expression, leading to reduced GPX4 activity and ferroptosis (21).
Molecular Blockers (RBP Mechanism)	LncRNAs bind to RBPs, blocking their interaction with downstream signaling pathways or mRNAs (22).	Lnc 00618 binds to LSH, reducing its recruitment to the SLC7A11 promoter and inhibiting ferroptosis (11).
Molecular Blockers (ceRNA Mechanism)	LncRNAs act as molecular sponges for miRNAs, regulating target gene expression (23).	Lnc RP11-89 sponges miR-129-5p, upregulating PROM2 and inhibiting ferroptosis in bladder cancer cells (10).
Bootstrap Mode	LncRNA-protein complexes localize to the DNA sequences of downstream genes, affecting their expression (24).	Lnc ASMTL-AS1 binds to U2AF2, stabilizing SAT1 mRNA expression and promoting ferroptosis in lung cancer cells (25).
Scaffolding Role	LncRNAs assemble multiple related transcription molecules, facilitating information interaction between different signaling pathways (26).	Lnc 00239 binds to KEAP1, inhibiting its binding to Nrf2 and stabilizing Nrf2 function, which in turn inhibits ferroptosis (27).

### 2.1 Signaling function

These mechanisms involve direct binding to DNA, proteins, and RNAs, thereby regulating the transcription and translation of cis- or transgenes (20). For example, lnc NEAT1 directly binds to and inhibits the expression of SLC7A11, leading to reduced GPX4 activity and subsequent ferroptosis (28). Lnc A2M-AS1 interacts with PCBP3, activating p38 and inhibiting the AKT-mTOR pathway, thereby promoting ferroptosis in pancreatic cancer cells (29). In hepatocellular carcinoma, LncRNA GABPB1-AS1 inhibits the translation of GABPB1, downregulating PRDX5 and reducing cellular antioxidant capacity, resulting in ferroptosis (30). In non-small cell lung cancer, MT1DP inhibits NRF2-mediated antioxidant effects through the miR-365a-3p/NRF2 axis, enhancing sensitivity to Erastin (31). POU6F1 binds to the promoter region of lncRNA-CASC2, promoting its transcription and inducing ferroptosis in gastric cancer cells (32). Lnc HCP5-132aa regulates GPX4 and suppresses ROS levels, inhibiting ferroptosis in triple-negative breast cancer (33). LINC02936 recruits SIX1 to the promoter region of the CP gene, upregulating CP expression and inhibiting ferroptosis in endometrial cancer (34).

### 2.2 Molecular blockers

LncRNAs also regulate ferroptosis through the ceRNA mechanism, acting as molecular sponges for miRNAs (35). For example, lnc RP11-89 inhibits ferroptosis in bladder cancer cells by sponging miR-129-5p and upregulating PROM2 (10). LINC UC.339 inhibits ferroptosis and promotes tumor proliferation in lung cancer cells through the UC.339/miR-339/SLC7A11 axis (36). LINC00336 acts as a sponge for MIR6852, regulating CBS expression and inhibiting ferroptosis in lung cancer cells (37). LINC01606 promotes SCD1 expression by interacting with miR-423-5p, inhibiting ferroptosis and promoting colorectal cancer progression (38). OIP5-AS1 acts as a sponge for miR-128-3p, increasing SLC7A11 expression and inhibiting ferroptosis in prostate cancer (12). NEAT1 promotes MIOX expression by sponging miR-362-3p, enhancing ferroptosis in hepatocellular carcinoma cells (39). LncRNAH19 enhances the anticancer effect of curcumin by sponging miR-19b-3p and inhibiting FTH1 expression (40). LncRNA PVT1 sponges miR-214-3p, reducing GPX4 expression and inducing ferroptosis in hepatocellular carcinoma cells (41).

### 2.3 Bootstrap mode

LncRNAs can also regulate the transcription of downstream genes by binding to proteins and localizing the complexes to the DNA sequences of the target genes (24). For example, lnc ASMTL-AS1 binds to U2AF2, stabilizing SAT1 mRNA expression and promoting ferroptosis in lung cancer cells (25). MAFG-AS1 binds to PCBP2, promoting the export of intracellular iron ions and inducing ferroptosis resistance in bladder uroepithelial carcinoma (42). FTX binds to FEN1, promoting its demethylation and increasing its expression, which in turn inhibits ferroptosis in oral squamous cell carcinoma cells (43).

### 2.4 Scaffolding role

Finally, lncRNAs can block downstream signaling pathways by binding to RNA-binding proteins (RBPs) (26). For example, LINC00618 binds to LSH, reducing its recruitment to the SLC7A11 promoter and inhibiting ferroptosis (11). TMEM44-AS1 binds to IGF2BP2, enhancing GPX4 stability and inhibiting ferroptosis in esophageal squamous cell carcinoma (44). P53RRA interacts with G3BP1, leading to P53 retention in the nucleus and promoting ferroptosis in lung cancer cells (45). SNAI3-AS1 interferes with the m6A-dependent recognition of Nrf2 by SND1, reducing Nrf2 stability and promoting ferroptosis in gliomas (46). SH3BP5-AS1 recruits IGF2BP2 to bind to VDAC2 mRNA, enhancing its stability and promoting ferroptosis in bladder cancer cells (47).

These mechanisms highlight the diverse roles of lncRNAs in regulating ferroptosis and provide insights into their potential as therapeutic targets for cancer treatment. **Table 1** summarizes the key mechanisms and examples.

## 3 LncRNAs regulate ferroptosis mechanisms through post-translational modifications

**Figure 2:**
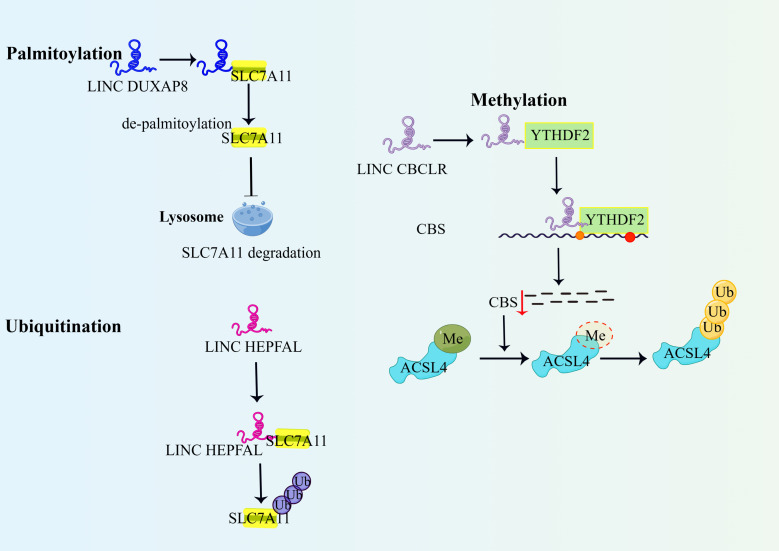
LncRNAs regulate ferroptosis through post-translational modification of binding proteins. 1)DUXAP8 promotes palmitoylation of SLC7A11 and prevents its lysosomal degradation, thereby enhancing the action of SLC7A11 and inhibiting ferroptosis. 2)LncRNA HEPFAL promotes ubiquitination of SLC7A11, decreases the stability of SLC7A11 protein, and promotes ferroptosis in hepatocellular carcinoma cells. 3)IncRNA-CBSLR recruits YTHDF2 protein and CBS mRNA to form the CBSLR/YTHDF2/CBS complex, which reduces CBS mRNA stability in an M6A-dependent manner. Reduced CBS expression reduced methylation of ACSL4 protein, and then ACSL4 was degraded via the ubiquitination-proteasome pathway. YTHDF2, YTH structural domain family protein 2; Me,methylation; ACSL4, Long-chain acyl-coenzyme A (CoA) synthase 4.

So far, a number of lncRNAs have been found to regulate the post-translational modifications of their RBPs through mechanisms such as phosphorylation, ubiquitination, methylation, and acetylation, which directly or indirectly affect the occurrence of ferroptosis by regulating protein degradation or production and affecting the expression level and activity of proteins (48). Several researchers have now demonstrated the regulatory role of lncRNAs in terms of ubiquitination, methylation, palmitoylation and acetylation. **Figure 2** summarizes some of the classical forms.

### 3.1 Ubiquitination

The ubiquitin protein-enzyasome pathway is a more general type of endogenous protein degradation in which proteins modified by ubiquitination regulate biological effects such as DNA damage repair and altered immune response by being degraded by the proteasome or by altering their activity (49). It was found that the LncRNA HEPFAL promotes ubiquitination of SLC7A11, leading to a decrease in GSH production, which in turn affects the activity of GPX4, ultimately leading to the development of ferroptosis (15). LINC00239 promotes CRC proliferation by interacting with Kelch-like ECH-associated protein 1 (Keap1), leading to instability of the Keap1/Nrf2 complex, and inhibition of Nrf2 ubiquitination to enhance its stability and promote colorectal cancer development. Importantly, Nrf2 also promotes transcription of LINC00239 in a positive feedback manner (27). LncFAL inhibits ex vivo anti-tumor activity by directly binding to and competitively attenuating FSP1-dependent Trim69 ubiquitination, reducing susceptibility to ferroptosis and inhibiting ex vivo anti-tumor activity (50).

### 3.2 Methylation

RNA methylation is a chemical modification phenomenon in which methyl adenines of RNA are selectively added with methyl groups catalysed by methyltransferases, the main form being m^6^A methylation. RNA methylation, as a ubiquitous post-transcriptional modification, plays a crucial role in regulating biological processes such as RNA transcription, splicing, structure, stability and translation (51). It was found that the LncRNA BDNF-AS regulates the transcription of the FBXW7 promoter by WDR5 methylation of its CpG island, and then FBXW7 regulates the expression of the VDAC3 protein by ubiquitination in gastric cancer cells. VDAC3 is the binding site for the ferroptosis-inducing drug, Erastin, and overexpression of VDAC3 increases sensitivity to Erastin (52). The results showed that when BDNF-AS was overexpressed, the expression level of VDAC3 protein increased. When VDAC3 protein is not degraded properly, the original dynamic homeostatic process is disrupted, and the abnormal increase in ion and energy metabolism promotes the proliferation, invasion and metastasis of tumor cells, leading to resistance to ferroptosis (52). Another study found that HIF-1 induced LncRNA-CBSLR to recruit YTHDF2 proteins and CBS mRNA to form the CBSLR/YTHDF2/CBS complex, which in turn reduced the stability of CBS mRNA in an m6A-dependent manner. Reduced CBS expression reduces methylation of ACSL4 protein. protects gastric cancer cells from ferroptosis in a hypoxic tumor microenvironment (53).

### 3.3 Palmitoylation

Palmitoylation refers to the post-translational modification of lipids and proteins and usually refers to the addition of 16-carbon palmitic acid to the cysteine of a protein via a thioester bond. Palmitoylation controls the association and transport of proteins to the cell membrane, thus playing a key role in protein function and cellular signal transduction (54). One study found that PCSK9 palmitoylation modification enhanced sorafenib resistance in hepatocellular carcinoma cells (55). Shi J et al. found that overexpression of LINC DUXAP8 in hepatocellular carcinoma cells could enhance the action of SLC7A11 by promoting palmitoylation of the XC-subunit SLC7A11 and preventing its lysosomal degradation, which in turn inhibited ferroptosis and enhanced the resistance of advanced hepatocellular carcinoma cells to sorafenib (56).

### 3.4 Acetylation

Acetylation is the chemical modification of the lysine portion of a protein by the selective addition of an acetyl group (-COCH3) in the presence of lysine acetyltransferase, which centres on the acetylation of lysine residues on ϵ-amino groups. Acetylation plays a significant role in regulating various functions of proteins, maintaining their stability, localising subcellularly, DNA replication and facilitating interactions between proteins (57, 58). Mi et al. found that LncRNA HOTAIRM1 inhibited radiotherapy-induced ferroptosis and promoted radiotherapy resistance in nasopharyngeal carcinoma cancer by interacting with the FTO protein, promoting FTO acetylation and enhancing its stability, which led to demethylation of the m6A of the CD44 precursor mRNA, which in turn affected the production of the splice isoform CD44V of CD44, and suppressed radiotherapy-induced ferroptosis (59). Patients with bladder urothelial carcinoma cells highly expressing the LncRNA MAFG-AS1 positively regulate MAFG gene expression by recruiting histone acetyltransferase p300 through cis-regulatory effects and promoting acetylation of the histone H3 lysine 27 site (H3K27ac) on the MAFG genome. This process forms a MAFG-antisense RNA 1 (AS1)/MAFG positive feedback regulatory loop, which inhibits ferroptosis and increases cellular chemoresistance to cisplatin (42). LncRNA SCARNA10 promotes the interaction of p53 with CREB-binding proteins by binding to the DNA-binding domain of p53 and increasing the level of p53 acetylation, which activates p53-mediated transcriptional activation and enhances the sensitivity of tumor cells to ferroptosis (60).

## 4 LncRNAs in ferroptosis of immune cells and tumor immunity

Ferroptosis, a unique form of programmed cell death characterized by iron-dependent lipid peroxidation, has gained considerable attention for its potential role in modulating the tumor immunomicroenvironment (61). Long non-coding RNAs (lncRNAs) have emerged as key regulators of ferroptosis in immune cells, thereby influencing their antitumor activities (62). This section will discuss the role of lncRNAs in regulating ferroptosis of T cells and macrophages, and their impact on the tumor immunomicroenvironment.

### 4.1 T cells

The ferroptosis status of T cells directly affects their anti-tumor activity (63). Recent studies have shown that lncRNAs play the role of “molecular switches” in T cell survival and function by regulating key pathways such as iron metabolism, antioxidant system and lipid peroxidation (64).

LINC00472 is highly expressed in tumor-infiltrating CD8^+^ T cells and inhibits ferroptosis through multiple mechanisms (65). First, it binds and stabilizes the mRNA of GPX4, a key inhibitor of ferroptosis, blocking its proteasomal degradation (66). It can also enhance the translational efficiency of SLC7A11 (cystine/glutamate reverse transporter) and maintain intracellular glutathione levels by recruiting the RNA-binding protein HuR (67). In addition, it was also able to deregulate the inhibitory effect of miR-30a-5p on ACSL4 (pro-iron death lipid metabolizing enzyme) by competitive adsorption of miR-30a-5p (68). In a melanoma model, LINC00472 knockdown resulted in a 50% reduction in T cell infiltration and accelerated tumor growth (69). In addition, GAS5 was able to be upregulated in a HIF-1α-dependent manner in hypoxic regions of the tumor (70). It can directly bind to the ferritin heavy chain (FTH1) promoter and inhibit its transcription, leading to free iron accumulation (71). Secondly, it promotes ferritin autophagy (ferritinophagy) by interacting with NCOA4 protein to release stored iron (72). It can also adsorb miR-137 through a ceRNA mechanism, which deregulates its inhibition of ALOX15 (lipoxygenase) and exacerbates lipid peroxidation (73). This mechanism leads to massive ferroptosis of T cells within the tumor, creating areas of immune desertification.

### 4.2 Macrophages

Ferroptosis status of macrophages profoundly affects their phenotypic transition and tumor microenvironment remodeling. Specific lncRNAs enable precise regulation through epigenetic modifications and signaling pathway cross-talk (74).

Macrophages are another critical component of the immune system and can be polarized into pro-inflammatory (M1) or anti-inflammatory (M2) phenotypes (75). LncRNAs have been shown to regulate macrophage ferroptosis, thereby impacting the tumor microenvironment (76). For example, by binding to LSH, LINC00618 inhibits the expression of SLC7A11, leading to increased lipid peroxidation and promoting ferroptosis in macrophages (11). This process can affect the polarization and function of macrophages in the tumor microenvironment, potentially enhancing antitumor immunity. Additionally, lncRNA HEPFAL can promote the ubiquitination of SLC7A11, reducing its stability and inducing ferroptosis in hepatocellular carcinoma cells, which can also impact the function of macrophages in the tumor microenvironment (15).

Notably, in tumor-associated macrophages (TAMs), FER1L4 maintains the M2 phenotype through a triple action. First, it acts as a “molecular sponge” for miR-214-3p, blocking its inhibition of GPX4 and maintaining antioxidant capacity (77). Second, it recruits DNMT3A to the Nrf2 promoter region and induces DNA methylation to inhibit activation of the Keap1-Nrf2 pathway (78). Third, binding to STAT6 protein enhanced M2 polarization driven by IL-4/IL-13 signaling (79). Analysis of clinical samples also showed that high FER1L4 expression was significantly associated with TAMs infiltration density and poor patient prognosis (80). NEAT1 is also known as a global regulator of iron metabolism. NEAT1 regulates the iron metabolism network through the formation of paraspeckles (81). It directs variable splicing of FTH1/FTL mRNA to generate iron storage-enhancing isoforms. It can also recruit HDAC1 to the TFRC (transferrin receptor) promoter, repressing its expression and reducing iron uptake (82). Formation of a liquid condensate by phase separation allows isolation of the iron-promoting death factor SAT1 (spermine/spermine N1-acetyltransferase) (83). A study noted that in a pancreatic cancer model, a NEAT1 inhibitor combined with a PD-1 antibody resulted in a 3-fold increase in tumor regression (84).

### 4.3 Effects of ferroptosis on the tumor immune microenvironment

Ferroptosis can promote the infiltration and activation of immune cells by releasing damage-associated molecular patterns (DAMPs) (85). These DAMPs, such as HMGB1 and S100 proteins, can bind to pattern recognition receptors on immune cells, promoting their infiltration and activation (86). For example, the release of HMGB1 from ferroptotic cells can bind to TLR4 on dendritic cells, promoting their maturation and enhancing their ability to present tumor antigens to T cells (87). The release of S100 proteins can bind to RAGE on natural killer (NK) cells, promoting their activation and enhancing their ability to kill tumor cells. Extracellular ATP activates NLRP3 inflammatory vesicles via P2X7 receptors, inducing IL-1β secretion and recruiting neutrophil infiltration (88). And preclinical studies have shown that this process enhances anti-CTLA-4 efficacy (89). In addition, 4-hydroxynonenal (4-HNE) can promote cytotoxic CD8^+^ T cell homing by modifying lysine residues of CXCL1 protein and enhancing its ability to bind to CXCR2 (90).

**Figure 3:**
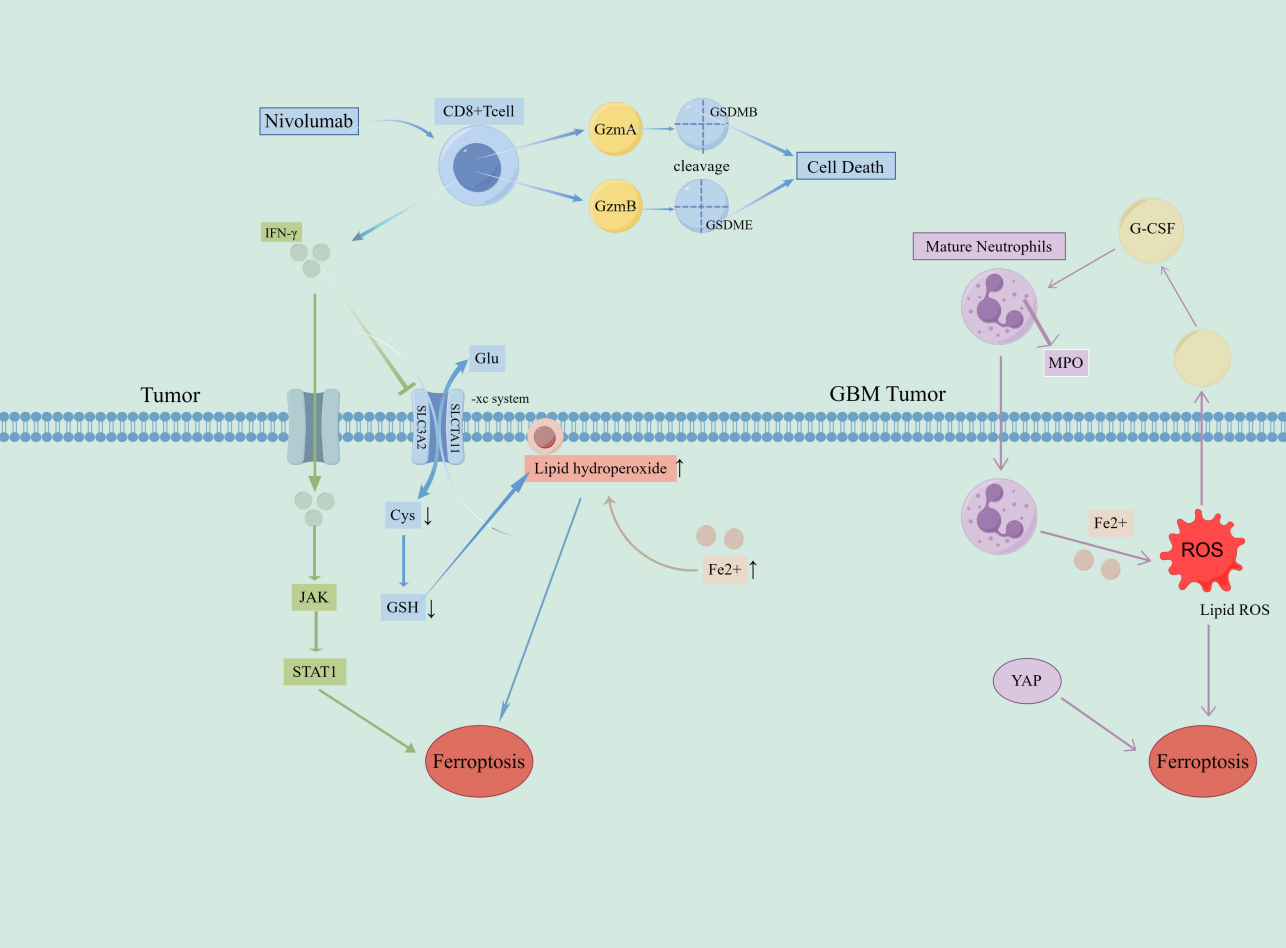
Effect of immune cells on ferroptosis in tumor cells. 1. Acting on cell surface receptors: CD8+ releases IFN-γ and acts on tumor cell surface receptor-xc system, reducing the production of antioxidant GSH in tumor cells, promoting lipid peroxide deposition in tumor cells, and inducing ferroptosis in tumor cells. 2. Intracellular action: IFN-γ enters cancer cells to activate the JAK/STAT1 pathway and induce ferroptosis. Mature neutrophils release MPO into tumor cells under the action of extracellular G-CSF, promote Lipid ROS, and induce ferroptosis in tumor cells. The content of Fe2+ and YAP protein in tumor cells increased, which promoted ferroptosis. GzmA, Granzyme A; GzmB, Granzyme B; GSDMB, Gasdermin B, GSDME, Gasdermin E, A class of proteins involved in pyroptosis and inflammation; SCL7A11, cystine-glutamate exchange system XC;Gys, Gystine; Glu, Glutamic acid; -; GSH, Glutathione; MPO, myeloperoxidase-containing granules; ROS, reactive oxygen species.

Notably, ferroptosis may promote immune escape under certain conditions (91). **Table 2** summarizes the major lncRNAs associated with ferroptosis and their roles in related cancers. For example, ferroptosis can reduce the expression of PD-L1 on the surface of tumor cells, decreasing their ability to suppress T cell activity and enhancing the antitumor immune response (100). Additionally, ferroptosis can increase the intracellular iron levels in tumor cells, inhibiting their proliferation and migration and reducing their ability to evade the immune system (101). Wang et al. reported that induction of ferroptosis in tumor cells significantly reduced their PD-L1 expression and enhanced the antitumor activity of T cells (102). **Figure 3** depicts the effect of iron death on immune cells.

**Table 2:** The major lncRNAs associated with ferroptosis and their roles in related cancers.

LncRNA Name	Role	Associated Cancer(s)	Mechanism
**LINC NEAT1**	Oncogenic	Lung Cancer, Melanoma	Binds SLC7A11 to inhibit ferroptosis; downregulates GPX4 activity (92) .
**LINC00336**	Oncogenic/Drug-Resistant	Lung Cancer	Sponges miR-6852 to stabilize CBS, reducing ROS and inhibiting ferroptosis (37).
**LINC HCP5-132aa**	Oncogenic	Triple-Negative Breast Cancer	Encodes HCP5-132aa to suppress ROS and inhibit ferroptosis via GPX4 (33).
**LINC00618**	Tumor-Suppressive	Lung Cancer	Binds LSH to inhibit SLC7A11 transcription, promoting ferroptosis (11).
**DUXAP8**	Drug-Resistant	Hepatocellular Carcinoma	Enhances SLC7A11 palmitoylation, stabilizing it to inhibit ferroptosis (56).
**HCG18**	Drug-Resistant	Hepatocellular Carcinoma	Sponges miR-450b-5p to upregulate GPX4, reducing sorafenib sensitivity (93).
**MT1DP**	Tumor-Suppressive	Non-Small Cell Lung Cancer	Downregulates NRF2 via miR-365a-3p, increasing Erastin-induced ferroptosis (31).
**P53RRA**	Tumor-Suppressive	Breast Cancer, Lung Cancer	Sequesters p53 in the nucleus to promote ferroptosis and apoptosis (45).
**LINC00239**	Oncogenic	Colorectal Cancer	Stabilizes NRF2 by binding Keap1, inhibiting ferroptosis (27).
**H19**	Tumor-Suppressive	Lung Cancer	Sponges miR-19b-3p to downregulate FTH1, enhancing curcumin-induced ferroptosis (40).
**PVT1**	Oncogenic/Drug-Resistant	Hepatocellular Carcinoma	Sponges miR-214-3p to upregulate GPX4, inhibiting ferroptosis (94).
**URB1-AS1**	Drug-Resistant	Hepatocellular Carcinoma	Promotes ferritin phase separation to reduce free iron, inhibiting ferroptosis (95).
**TMEM161B-AS1**	Drug-Resistant	Glioma	Upregulates FANCD2/CD44 via miR-27a-3p to suppress ferroptosis (96).
**HAND2-AS1**	Tumor-Suppressive	Hepatocellular Carcinoma	Activates TLR4/NOX2/DUOX2 axis to induce ferroptosis and reverse lenvatinib resistance (97).
**FTX**	Oncogenic	Oral Squamous Cell Carcinoma	Recruits TET2 to demethylate FEN1 promoter, suppressing ACSL4 and ferroptosis (98).
**HEPFAL**	Tumor-Suppressive	Hepatocellular Carcinoma	Promotes SLC7A11 ubiquitination, reducing GSH and inducing ferroptosis (15).
**PCAT1**	Drug-Resistant	Prostate Cancer	Stabilizes c-Myc to upregulate SLC7A11, inhibiting docetaxel-induced ferroptosis (99).

### 4.4 Breakthroughs in therapeutic strategies targeting the ferroptosis - immunity axis

Ferroptosis inducers can enhance the efficacy of immunotherapy by promoting the infiltration and activation of immune cells (85). Ferroptosis inducers (e.g. Erastin) selectively remove immunosuppressive cells from tumors by depleting GSH and disarming the antioxidant defense of Tregs (103). Lipid peroxides (e.g., LPO) activate CD36 scavenger receptors in DCs and promote tumor antigen cross-presentation (104). In addition, 8-OHdG released from iron death activates type I interferon response through the STING pathway, which can enhance NK cell killing activity (105). The combination of ferroptosis inducers and immune checkpoint inhibitors has been shown to enhance the antitumor immune response in preclinical and clinical studies (106). In a study by Rosato et al., the combination of ferroptosis inducers and anti-PD-1 antibodies significantly enhanced the antitumor immune response and improved the survival rates of mice with established tumors. In a PDX model of triple-negative breast cancer, application of the GPX4 inhibitor ML162 in combination with anti-PD-1 therapy increased the patient complete remission rate from 15% to 65% (107). In another trial in advanced melanoma phase I (NCT05154227), the iron carrier drug Ciclopirox combined with a CTLA-4 inhibitor resulted in a 48% objective remission rate with manageable toxicity (108).

**Figure 4:**
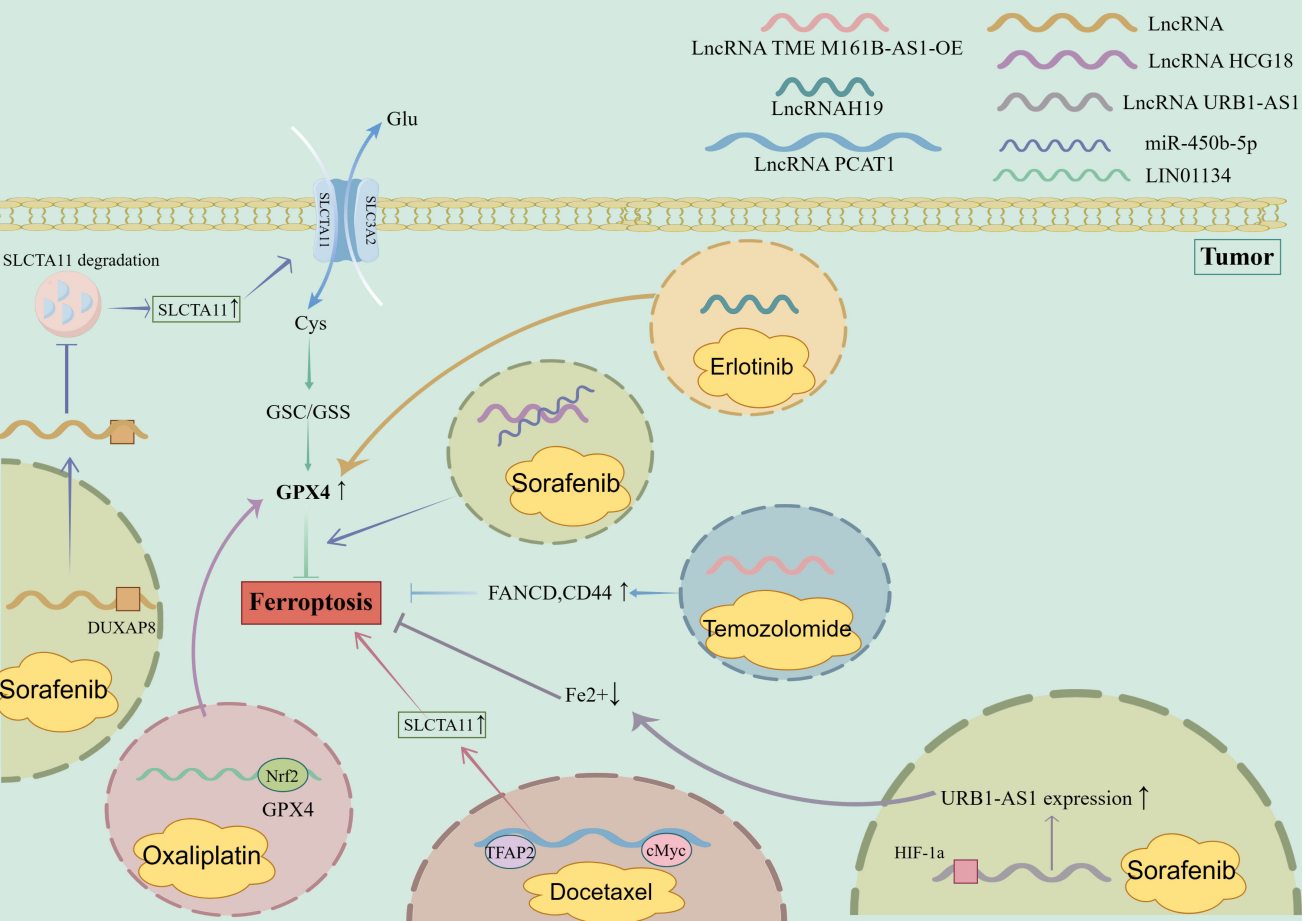
Role of LncRNA in promoting ferroptosis in chemoresistant tumor cells. 1. Sorafenib-resistant tumor cells (1): LncRNA HCG18 binds to miR-450B-5p to promote the inhibition of ferroptosis by GPX4; (2) LncRNA URB1-As interacted with HIF-1a to increase the expression of URB1-AS1, reduce the content of free iron in cells, and inhibit ferroptosis. (3) LncRNA binds to DUXAP8 to inhibit the degradation of SLCTA11 and promote the inhibition of ferroptosis. 2. Oxaliplatin-resistant tumor cells: Nrf2 binds to the LIN01134 GPX4 promoter position, induces increased GPX4 expression, and inhibits ferroptosis. 3. Docetaxel-resistant tumor cells: LncRNA PCAT1, TFAP2 bind to cMyc, enhance the expression of SLCTA11, and inhibit ferroptosis. 4. Temozolomide-resistant tumor cells: Overexpression of LncRNA TME M161B-AS1 leads to increased expression of FANCD and CD44 and inhibits ferroptosis. 5. Erlotinib drug-resistant tumor cells: LncRNAH19 binds to β-elemene, increases the expression of GPX4, and inhibits ferroptosis. DUXAP8, Deluxe Universal Auxiliary Power 8; Nrf2, Nuclear factor erythroid 2-related factor 2; TFAP2, Transcriptional regulation by the AP-2; cMyc, Myc proto-oncogene protein, HIF-1a, Hypoxia Inducible Factor-1; FANCD, FA Complementation Group D.

Specific lncRNAs can serve as potential targets for immunotherapy by regulating the ferroptosis of immune cells (109). **Figure 4** depicts role of lncRNAs in promoting ferroptosis in chemoresistant tumor cells. For example, targeting lncRNA H19 has been shown to enhance the efficacy of immunotherapy by promoting the ferroptosis of cancer cells and enhancing the antitumor activity of T cells (110). Additionally, targeting lncRNA PVT1 has been shown to enhance the efficacy of immunotherapy by promoting the ferroptosis of cancer cells and enhancing the antitumor activity of T cells (111). Zhang et al. reported that targeting lncRNA H19 significantly enhanced the antitumor effects of immunotherapy in a mouse model of hepatocellular carcinoma (112).

In conclusion, lncRNAs play crucial roles in regulating the ferroptosis of immune cells and impacting the tumor immunomicroenvironment. By understanding the mechanisms by which lncRNAs regulate ferroptosis and their interplay with the immune system, researchers can develop new therapeutic strategies to enhance the efficacy of cancer immunotherapy. Cross-regulation of ferroptosis and tumor immunity constitutes a multidimensional signaling network, and lncRNAs, as the “molecular hub” of this network, provide a new perspective for overcoming immunotherapy resistance. In the future, we need to analyze the dynamic regulatory maps through interdisciplinary cooperation (e.g., single-cell multi-omics, spatial metabolic imaging), and ultimately realize the breakthrough from mechanism research to clinical translation.

## 5 Conclusions and outlook

Exploring the mechanism of tumor development and finding effective treatments for tumors has been a hot spot in current molecular biology research (113). However, with the occurrence of chemotherapy resistance, radiotherapy resistance and the biological process of tumor immune escape, tumor patients enter the recurrence stage after a short period of remission until death (114). This requires a more refined study of the mechanism of tumorigenesis and the mechanism of tumor drug resistance. With the completion of the Human Genome Project, it was discovered that only 2% of the genome codes for proteins, 85% of human genes are transcriptionally controlled, and LncRNAs account for 98% of these non-coding RNAs (115). LncRNAs were initially thought to be by-products of RNA polymerase II transcription, and to be the ‘noise’ of genome transcription. LncRNAs were initially thought to be by-products of RNA polymerase II transcription and ‘noise’ of genome transcription, with no biological function. With the advancement of research, it has been found that LncRNAs are involved in processes including transcription, post-transcriptional and translational regulation, epigenetic processes, immune response, differentiation, imprinting, maintenance of pluripotency, cell cycle regulation, apoptosis, and cellular senescence (116). Since LncRNAs modulate cancer type specificity through various pathways, they are attractive targets for selective therapeutic interventions.

Ferroptosis is a novel form of programmed cell death dependent on iron ions and distinct from apoptosis, autophagy and necrosis. In this paper, we provide a systematic review of the role of ferroptosis in modulating chemotherapy-resistant cancer cell sensitisation, modulating immune escape of tumor cells and the current application of nanomaterials in inducing ferroptosis in tumor cells, and review the mechanism of the role of LncRNAs in cancer progression and ferroptosis regulation, which can help to further our understanding of the pathogenesis of cancer. Targeting these key LncRNAs using nanomaterials may lead to the discovery of new diagnostic and therapeutic approaches to inhibit the growth of malignant tumors.

Despite such a large system of LncRNAs playing a huge role in regulating the expression of ferroptosis-related genes, there are still some challenges to be overcome, which are accompanied by significant opportunities. First, the tumor microenvironment is a very complex environment composed of hypoxia, tumor inflammation, and immune escape, etc. The tumor microenvironment varies at different stages of tumorigenesis, so the LncRNAs regulating the ferroptosis process may be a group of LncRNAs, which interact with each other, making the tumor cells insensitive to the induction of ferroptosis, which makes the study more difficult (117). Secondly, ferroptosis is a double-edged sword; ferroptosis can be used both as a treatment for cancer and may also induce cancer. In the process of inducing ferroptosis in the organism, although it can effectively kill tumor cells, it may also induce the death of cells with tumor-killing viability such as CIK cells, NK cells and CD cells. Therefore, precise knockdown or overexpression of ferroptosis-related genes in cancer-causing cells in the organism to induce ferroptosis is a challenge that needs to be solved for the application of ferroptosis to treat tumors (118). Third, it was found that not all cancer cells are sensitive to ferroptosis. However, ferroptosis is a complex cell death process that is co-regulated by a variety of factors, and elevated ROS levels as well as intracellular Fe^2+^ alone cannot be used as criteria for determining ferroptosis. Because of the different triggering mechanisms and the different sensitivity to different ferroptosis regulators, it may be more accurate to determine whether such cancer cells are sensitive to ferroptosis by selecting multiple pathways of ferroptosis inducers during the initial study (119). Fourth, certain chemotherapeutic agents are designed and applied to inhibit the growth and spread of tumor cells by inducing ferroptosis (120). However, cancer cells may become resistant to these chemotherapeutic agents through a variety of mechanisms, such as accelerated drug metabolism by increasing the activity of drug-metabolising enzymes or altering the expression of membrane transporter proteins, altering the target site of drug action through genetic mutations, altering the target molecule of drug action so that the drug does not bind efficiently, or decreasing the susceptibility to ferroptosis through activation of the intracellular antioxidant defence system (121). The development of drug resistance not only reduces the efficacy of chemotherapeutic drugs, but may also lead to tumor recurrence and treatment failure. Increased drug resistance not only reduces the efficacy of chemotherapeutic drugs, but may also trigger tumor recurrence as well as treatment failure. Therefore, in order to improve the induction of ferroptosis, inhibit the activation of drug resistance mechanisms, and precisely target ferroptosis-related gene expression using epigenetic modulators, in-depth scientific investigations are still needed. Fifth, ferroptosis is a metabolism-related cell death process, and intracellular ferroptosis is a process closely related to cellular metabolism, and changes in tiny molecules such as intracellular iron, selenium, oxygen, cysteine, glutathione, polyunsaturated fatty acids, and vitamin E may all play a key role in regulating ferroptosis (122). However, cancer has a very complex metabolic process, and understanding the metabolic process of cancer and then reducing the intake or increasing the rapid clearance of the relevant substances in cancer therapy can be beneficial in increasing the sensitivity of cancer cells to ferroptosis.

**Figure 5:**
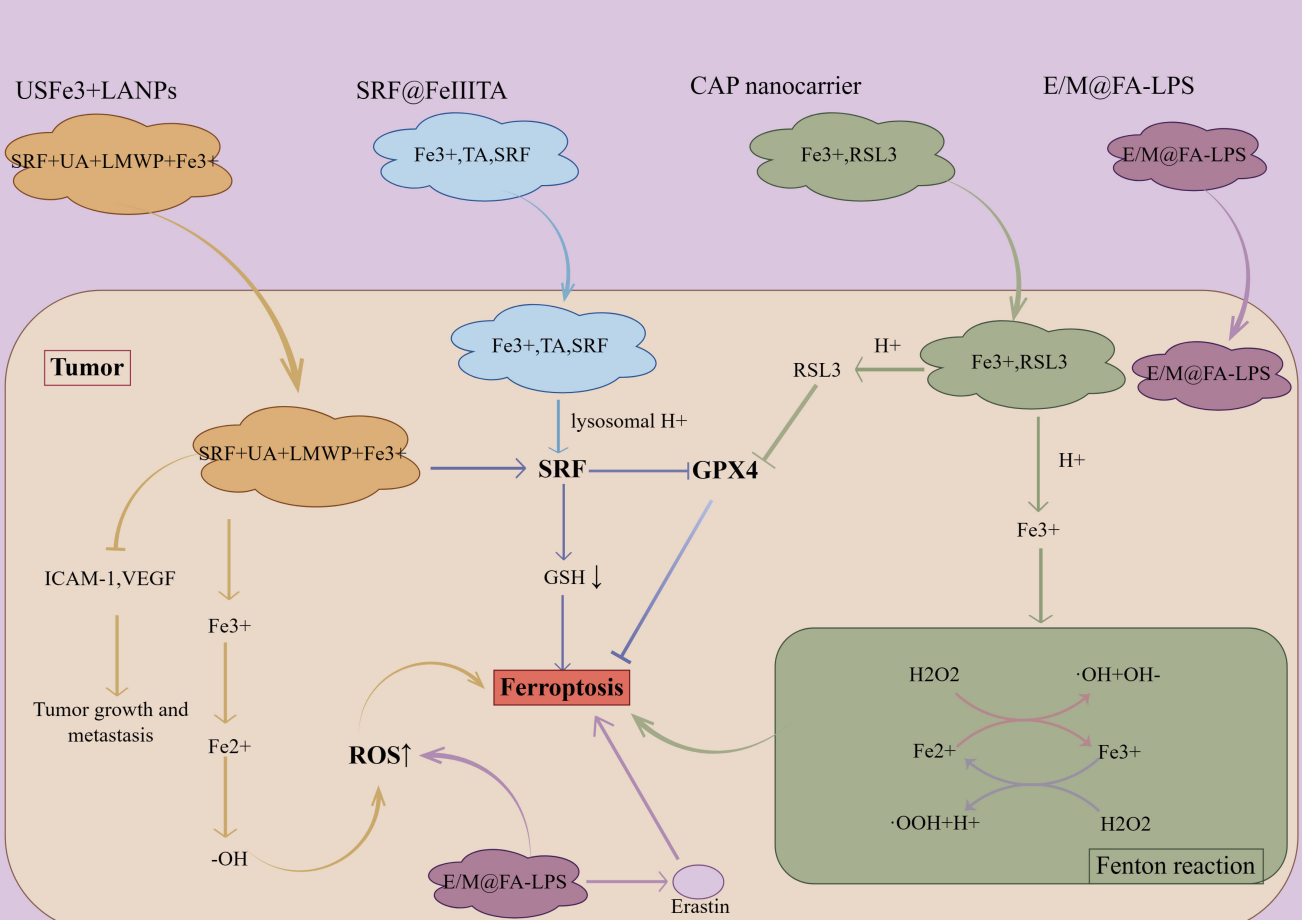
The role of nanomaterials in inducing ferroptosis in tumor cells. 1. Both USFe3+ LANPs nanoparticle materials and CAP nanocarrier release Fe3+ in tumor cells, and generate -OH and Fe2+ through Fenton reaction, which promotes ferroptosis of tumor cells, while the former inhibits the growth and metastasis of tumors by inhibiting the expression of ICAM-1 and VEGF. The latter releases the GPX4 inhibitor RSL3, inhibits GPX4, and promotes ferroptosis. 2.SRF@FeIIITA nanomaterials release SRF in tumor cells in the lysosomal H+ environment to induce ferroptosis. 3. E/M@FA-LPS nanomaterials promote ROS in tumor cells and induce ferroptosis. SRF, Sorafenib; UA, Ursolic acid; LMWP, Low molecular weight protamine; ICAM-1, Intercellular cell adhesion molecule-1; VEGF, Vascular endothelial growth factor; TA, Tannic acid.

With the understanding of the relationship between ferroptosis and LncRNAs, combining ferroptosis with other tumor cell death types would improve tumor outcomes. Current studies have shown that nanomaterials can not only deliver ferroptosis inducers and LncRNAs, but also induce the onset of ferroptosis using their own physical properties (**Figure 5**). Although studies targeting the induction of ferroptosis by LncRNAs are still in their infancy, such a large transcriptional system also offers great potential for cancer therapy, and multidisciplinary collaboration is expected to advance ferroptosis research.

## Glossary

4-HNE: 4-Hydroxynonenal
ACSL4: Acyl-CoA Synthetase Long Chain Family Member 4
ALOX15: Arachidonate 15-Lipoxygenase
ATP: Adenosine Triphosphate
CBS: Cystathionine Beta-Synthase
ceRNA: Competing Endogenous RNA
CXCL1: C-X-C Motif Chemokine Ligand 1
CXCR2: C-X-C Chemokine Receptor Type 2
DAMPs: Damage-Associated Molecular Patterns
DC: Dendritic Cell
DNMT3A: DNA Methyltransferase 3A
ELAVL1: ELAV-Like RNA-Binding Protein 1
FANCD: Fanconi Anemia Complementation Group D
FSP1: Ferroptosis Suppressor Protein 1
FTH1: Ferritin Heavy Chain 1
FTL: Ferritin Light Chain
GABPB1: GA-Binding Protein Transcription Factor Beta Subunit 1
GPX4: Glutathione Peroxidase 4
GSH: Glutathione
H3K27ac: Histone H3 Lysine 27 Acetylation
HIF-1α: Hypoxia-Inducible Factor 1-Alpha
HMGB1: High Mobility Group Box 1
ICAM-1: Intercellular Adhesion Molecule-1
IL-1β: Interleukin-1 Beta
Keap1: Kelch-Like ECH-Associated Protein 1
LPO: Lipid Peroxides
LSH: Lymphoid-Specific Helicase
m6A: N6-Methyladenosine
miRNA: MicroRNA
MPO: Myeloperoxidase
NEAT1: Nuclear Paraspeckle Assembly Transcript 1
NK细胞: Natural Killer Cell
NRF2: Nuclear Factor Erythroid 2-Related Factor 2
PD-L1: Programmed Death-Ligand 1
RBPs: RNA-Binding Proteins
ROS: Reactive Oxygen Species
S100: S100 Calcium-Binding Protein
SAT1: Spermidine/Spermine N1-Acetyltransferase 1
SCD1: Stearoyl-CoA Desaturase 1
SLC7A11: Solute Carrier Family 7 Member 11
STING: Stimulator of Interferon Genes
TAMs: Tumor-Associated Macrophages
TFRC: Transferrin Receptor
TLR4: Toll-Like Receptor 4
Tregs: Regulatory T Cells
VEGF: Vascular Endothelial Growth Factor
YAP: Yes-Associated Protein
